# A method for constructing word sense embeddings based on word sense induction

**DOI:** 10.1038/s41598-023-40062-3

**Published:** 2023-08-09

**Authors:** Yujia Sun, Jan Platoš

**Affiliations:** 1 Department of Computer Science, Technical University of Ostrava, 17. Listopadu 2172/15, 70800 Ostrava-Poruba, Czech Republic; 2 Institute of Network Information Security, Hebei GEO University, No. 136 East Huai΄an Road , Shijiazhuang, 050031 Hebei China

**Keywords:** Computer science, Information technology

## Abstract

Polysemy is an inherent characteristic of natural language. In order to make it easier to distinguish between different senses of polysemous words, we propose a method for encoding multiple different senses of polysemous words using a single vector. The method first uses a two-layer bidirectional long short-term memory neural network and a self-attention mechanism to extract the contextual information of polysemous words. Then, a *K*-means algorithm, which is improved by optimizing the density peaks clustering algorithm based on cosine similarity, is applied to perform word sense induction on the contextual information of polysemous words. Finally, the method constructs the corresponding word sense embedded representations of the polysemous words. The results of the experiments demonstrate that the proposed method produces better word sense induction than Euclidean distance, Pearson correlation, and KL-divergence and more accurate word sense embeddings than mean shift, DBSCAN, spectral clustering, and agglomerative clustering.

## Introduction

Polysemy is an inherent feature of natural language. Word sense induction (WSI) is a fundamental task in natural language processing (NLP). It considers the contextual information of polysemous words as a representation of word senses and utilizes clustering techniques to inductively analyze contextual information. It is thus of paramount significance to understand polysemous words and the representation of word sense; as such, this is an essential step towards developing an effective method of acquiring word sense knowledge to understand polysemous words.

Word embedding, a general term for language modeling and representation learning in NLP, has become a standard technology in this domain. It is also a technology that converts words represented in natural language into vector or matrix representations that computers can process. Word embedding models can be divided into two types: static word embedding models and contextual word embedding models. Static word embedding models, such as Word2Vec^1^ and GloVe^2^, use a single word vector per word, and polysemous words are no exception. In polysemous words, a word vector comprises several senses, which weakens the sense of each polysemous word to some degree. Therefore, it is impractical to use one representation to express different senses of the same word.

Contextual word embedding models, such as ELMo^3^ and BERT^4^, can obtain highly contextualized word representations from language models based on specific inputs (words are represented differently depending on their context) and have been demonstrated to be highly effective in a variety of NLP tasks. Due to the low precision of semantic expression in a specific field, or the limitations of applications with high real-time requirements, such as recommendation systems, information retrieval, and other tasks, there are problems with high latency and high model scale in these models.

NLP tasks suffer from word embedding due to the ubiquitous presence of polysemous words. To address the possible problems mentioned above, word sense embeddings has emerged as a new area of research in NLP. To convert coarse-grained word embeddings into fine-grained sense embeddings, each sense of a word is represented by a separate vector, and the different senses of words are distinguished by different word sense embeddings. As a result, we are now able to avoid the problem of the senses being confused.

In this paper, we propose a method for constructing word sense embeddings for polysemous words using a WSI task. The main contributions of this paper are as follows:

- Our proposed method utilizes a two-layer bidirectional long short-term memory (Bi-LSTM) neural network and a self-attention mechanism to represent each instance of the input as a contextual vector, which provides a deep representation of the contextual features of polysemous words for subsequent word sense induction.
- A *K*-means algorithm, which has been improved by optimizing the density peaks clustering (DPC) algorithm based on cosine similarity to perform WSI for each instance, dynamically perceives the word sense and constructs the word sense embeddings. Every cluster in the clustering result represents a word sense, and the cluster center represents the word sense embedding for the polysemous word.

Finally, we constructed word sense embeddings for 10 polysemous words that were part of the SemEval-2007 Task 17: English Lexical Sample^5^. The experimental results demonstrate that the method in this paper can provide high-quality word sense embeddings.

## Related work

The field of word sense embeddings can be divided into two main approaches depending on how the sense distinctions are defined: (1) unsupervised, where senses are learned directly from text corpora; and (2) knowledge-based, in which senses are linked to a predefined sense inventory by applying an underlying knowledge resource.

- Unsupervised. In these representation models, sense distinctions are derived from the analysis of text corpora. This paradigm is closely related to WSI. In this vein, Panigrahi et al.^6^ presented an unsupervised method for generating interpretable Word2Sense word embeddings. This LDA-based generative model can be extended to refine the representation of polysemous words within a short context, allowing the embeddings to be used in context-sensitive applications. Li et al.^7^ proposed an adaptive cross-contextual word embedding method based on topic modelling that can learn an unlimited number of tailored word embeddings for a polysemous word in different contexts. Jr et al.^8^ developed a sense-aware framework capable of processing multi-sense word information without the need for annotated data. This particular framework provides context representations without ignoring word order information or long-term dependencies. Chang et al.^9^ outlined a novel embedding method for a text sequence (i.e., a phrase or a sentence), in which the sequence is represented by a set of multi-mode codebook embeddings intended to capture different semantic facets. Manchanda et al.^10^ extended the skip-gram model by clustering the occurrences of the multi-sense words and accounting for their diversity in the contexts of Word2Vec to obtain accurate and efficient vector representations for each sense. A method of obtaining multi-sense word embedding distributions based on an asymmetric Kullback–Leibler divergence energy function was developed by Jayashree et al.^11^ for capturing textual entailment with a variant of the max-margin objective. Some studies^12,13^ used weighting scheme and graph attention network, optimize word and semantic representation.
- Knowledge-based. This technique represents word senses as defined in lexical knowledge bases (LKBs). Generally, LKBs are resources that index and classify words according to their properties and senses. LKBs have been successfully applied in a wide variety of language processing applications. The most common LKBs are WordNet^14^, Wikipedia^15^, BabelNet^16^, and ConceptNet^17^. Scarlini et al.^18^ proposed a semi-supervised method for producing sense embeddings that are comparable to contextualized word vectors for lexical meanings within a lexical knowledge base. Oele et al.^19^ proposed a simple, knowledge-based WSD method that employs word and sense embeddings to evaluate the similarity between the gloss of a sense and its context. Niu et al.^20^ adopted a knowledge-based approach and employed an attention scheme to identify word senses based on the contexts in HowNet, with a preference for semantic information. The sense knowledge graph was integrated into a single word embedding by Fang et al.^21^ to generate multi-sense embeddings and learn the relationships between sense embeddings and word embeddings by relying on sense-word interactions. Loureiro et al.^22^ outlined a general framework for learning sense embeddings with transformers and evaluated them extensively. Hedderich et al.^23^ developed a method for selecting multi-sense vector embeddings from the input sequence using an attention mechanism. Ruas et al.^24^ proposed a multi-sense embedding system called MSSA, which was applied to a traditional Word2Vec implementation, resulting in more robust multi-sense embeddings with minimal hyperparameter tuning. Zhou eta al.^25^ outlined a method for extracting sense-related information from contextualized embeddings and injected it into static embeddings to produce sense-specific static embeddings.

## Methodology

**Figure 1:**
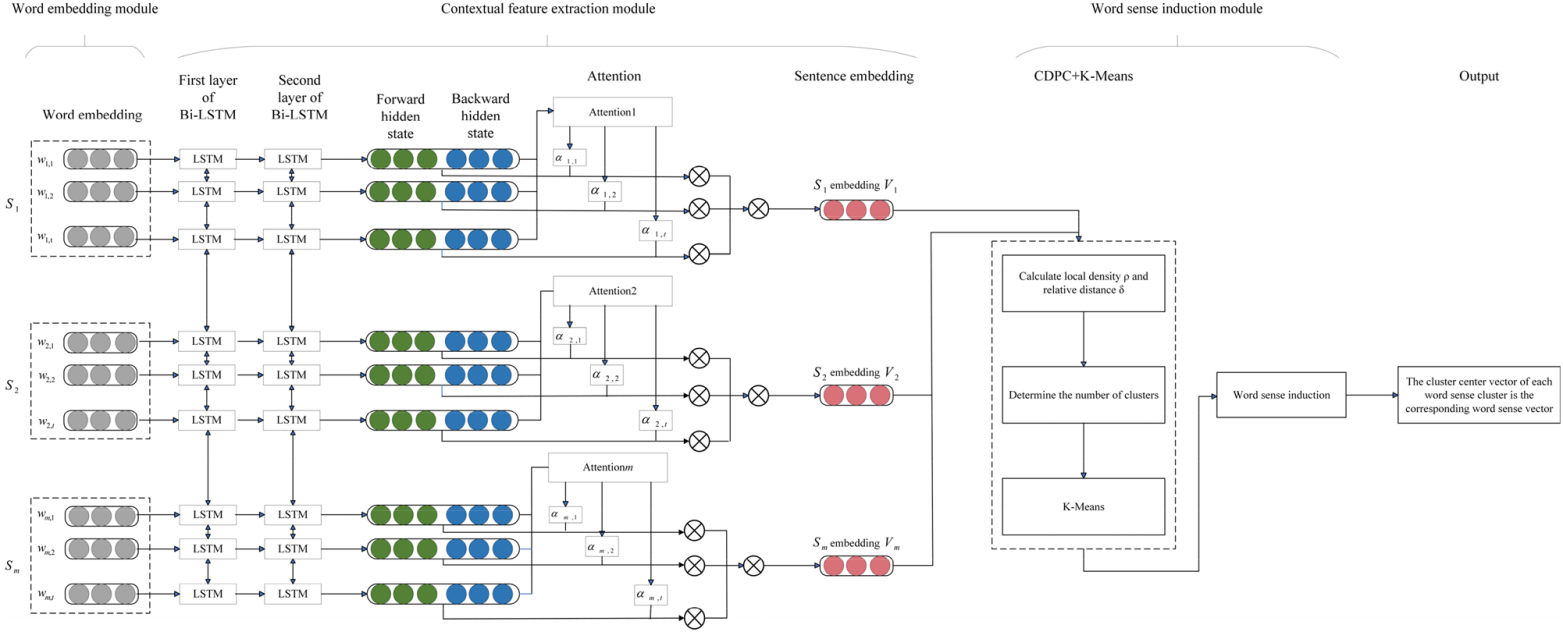
Architecture of the proposed method.

Our method is based on representation learning and utilizes a two-layer Bi-LSTM and attention mechanism, followed by WSI. The model is based on a *K*-means algorithm that was improved by optimizing the DPC clustering algorithm based on cosine similarity, resulting in polysemous word sense embeddings. The method we proposed in our research is illustrated in Fig. 1. As shown in the figure, the method is comprised of three modules: word embedding, contextual feature extraction, and WSI. Each module involves multiple steps.

### Word embedding module

We cleaned the corpus before the word embedding module, removing punctuation and special characters and retaining those parts that can be extracted as semantic information^26–28^. The word embedding module in this paper transformed the input sequence into vector form, and our approach used pre-trained 100-dimensional GloVe^2^ embeddings to map each word into a 100-dimensional word vector to obtain the corresponding sequence of word vectors. For a sentence of length *t* $\left\{{W}_{1},{W}_{2},\dots ,{W}_{t}\right\}$, the module then executes a word vector mapping on the word sequence through the pre-trained word vector model GloVe, resulting in a word vector matrix $\left\{{X}_{1},{X}_{2},\dots ,{X}_{t}\right\}$, whose size is $t*d$, whereby *d* is the dimension of the word vector.

### Contextual feature extraction module

#### Two-layer Bi-LSTM neural network

In general, when people read sentences with polysemous words, they determine their sense based on the context of the words. Therefore, we consider the context of polysemous words to be a feature of the sense induction process. The contextual feature extraction module is intended to capture the contextual information surrounding polysemous words through the use of two-layer Bi-LSTM and attention mechanisms to encode polysemous words and contexts and to extract deep feature vectors that contain contextual information. By using a two-layer Bi-LSTM approach as a basis for representing contextual information for polysemous words and assigning different attention weights to the feature vectors produced by Bi-LSTM, the correlation between contextual words and attention vectors can be determined, and the weighted sum of the correlation and the contextual words used as the contextual embedding effectively capture the semantic information related to polysemy words in depth.

**Figure 2:**
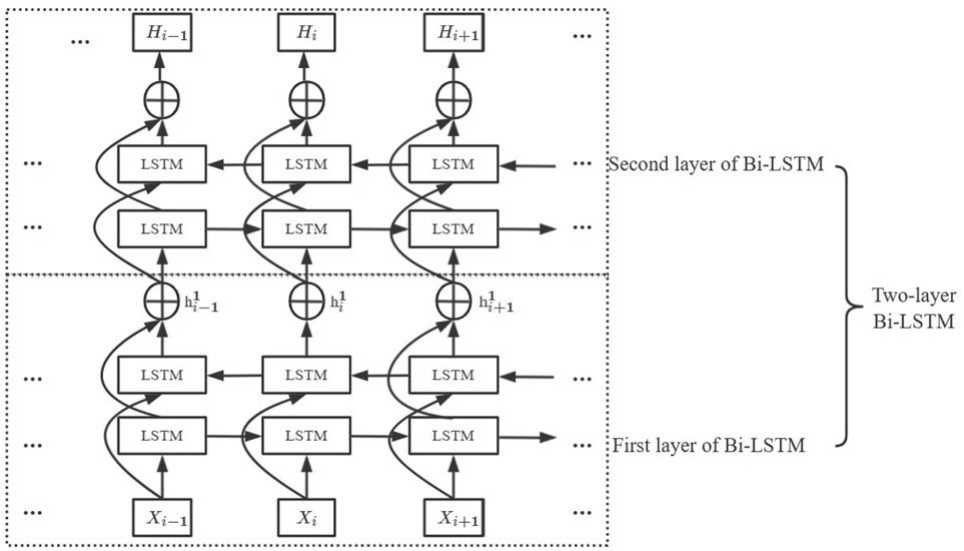
Architecture of two-layer Bi-LSTM networks.

As a solution to the problem of RNN gradient exploding, Hochreiter et al.^29^ developed a long short-term memory neural network (LSTM). This network introduces a gate mechanism structure to determine whether previous information should be lost or retained, thereby reducing the long-range dependency problem to some extent. LSTMs are capable of efficiently modelling the sequential features of text and can retain a certain amount of contextual information. They are most frequently used for data classification challenges, such as natural language translation, intelligent speech, and text classification. It should be noted that LSTM only takes into account the historical information of the current word in the text and ignores any future data. Hence, our method relies on Bi-LSTM instead of LSTM, which solves the gradient problem and incorporates contextual semantic information. Bi-LSTM is a hybrid of a forward LSTM and backward LSTM, which can learn forward semantic information and backward semantic information from text, respectively. Then, the forward and backward LSTMs encode the input data $\left\{{X}_{1},{X}_{2},\dots ,{X}_{t}\right\}$, and the forward and backward feature vector matrices are derived from $\overrightarrow{{h}_{t}}$ and $\overleftarrow{{h}_{t}}$. Concatenating of $\overrightarrow{{h}_{t}}$ with $\overleftarrow{{h}_{t}}$ produces the hidden state output of Bi-LSTM ${h}_{t}$ , as shown in Eqs. (1)–(3).

1 $$\overrightarrow{{h}_{t}}=\overrightarrow{LSTM}\left(\left[{X}_{1},{X}_{2},\dots ,{X}_{t}\right]\right)$$

2 $$\overleftarrow{{h}_{t}}=\overleftarrow{LSTM}\left(\left[{X}_{1},{X}_{2},\dots ,{X}_{t}\right]\right)$$

3 $${h}_{t}=\left[\overrightarrow{{h}_{t}};\overleftarrow{{h}_{t}}\right]$$

where $\left[\overrightarrow{{h}_{t}};\overleftarrow{{h}_{t}}\right]$ denotes concatenate $\overrightarrow{{h}_{t}}$ with $\overleftarrow{{h}_{t}}$. Compared with a single-layer Bi-LSTM neural network, a two-layer Bi-LSTM neural network can learn more complex feature representations and has enhanced learning capabilities. This two-layer Bi-LSTM model uses two Bi-LSTM layers, with the output of the first layer being used as an input to the second layer, thereby improving the depth and capacity of the LSTM network and its ability to represent features. Figure 2 below illustrates a two-layer Bi-LSTM network.

First, the input to the first Bi-LSTM layer is the output of the word embedding module $\left\{{X}_{1},{X}_{2},\dots ,{X}_{i},\dots ,{X}_{t}\right\}$. The first Bi-LSTM layer transmits the hidden state, where $i$ represents the $ith$ word, and the parameters ${w}_{1}$ and ${b}_{1}$ are shared between this layer. The output of the first Bi-LSTM layer is a concatenated sequence of features obtained from the hidden state of the forward LSTM and the hidden state of the backward LSTM as the input to the second Bi-LSTM layer. In the second Bi-LSTM layer, the forward and backward hidden states of the text are concatenated to generate a feature vector representation of the text. The vector ${h}_{i}$ represents the deep-level dependencies implicit in the text, which are crucial to improving the classification accuracy. The hidden state ${h}_{i}^{1}$ of the first layer can be expressed as follows:

4 $$\overrightarrow{{h}_{i}^{1}}=\overrightarrow{LSTM}\left({X}_{i},\overrightarrow{{h}_{i-1}^{1}}\right)$$

5 $$\overleftarrow{{h}_{i}^{1}}=\overleftarrow{LSTM}\left({X}_{i},\overleftarrow{{h}_{i-1}^{1}}\right)$$

6 $${h}_{i}^{1}=\left[\overrightarrow{{h}_{i}^{1}};\overleftarrow{{h}_{i}^{1}}\right]$$

where $\left[\overrightarrow{{h}_{i}^{1}};\overleftarrow{{h}_{i}^{1}}\right]$ denotes concatenate $\overrightarrow{{h}_{i}^{1}}$ with $\overleftarrow{{h}_{i}^{1}}$.

In the second layer, the final output of the two-layer Bi-LSTM ${H}_{i}$ is calculated as follows:

7 $$\overrightarrow{{h}_{i}^{2}}=\overrightarrow{LSTM}\left({h}_{i}^{1},\overrightarrow{{h}_{i-1}^{2}}\right)$$

8 $$\overleftarrow{{h}_{i}^{2}}=\overleftarrow{LSTM}\left({h}_{i}^{1},\overleftarrow{{h}_{i-1}^{2}}\right)$$

9 $${H}_{i}=\left[\overrightarrow{{h}_{i}^{2}};\overleftarrow{{h}_{i}^{2}}\right]$$

where $\left[\overrightarrow{{h}_{i}^{2}};\overleftarrow{{h}_{i}^{2}}\right]$ denotes concatenate $\overrightarrow{{h}_{i}^{2}}$ with $\overleftarrow{{h}_{i}^{2}}$, ${H}_{i}\in {R}^{{2d}_{h}}$, ${d}_{h}$ is the size of the LSTM hidden layer. While the Bi-LSTM unit emphasizes recent inputs when extracting feature information, it does not capture relatively distant information related to the sense of polysemous words in the text. The attention mechanism compensates for this shortcoming. Attention mechanisms have been demonstrated to increase the correlation between data by introducing supplementary variables that are trainable. Furthermore, they can highlight specific data segments to assist the network in capturing stage-specific information. For these reasons, the attention mechanism was applied to the hidden state vector of the two-layer Bi-LSTM as a means of further optimizing the contextual information.

#### Self-attention mechanism

The attention mechanism was first introduced by Mnih et al.^30^, who designed the model to pay specific attention to specific regions or individuals during training. The purpose of this design was to enable weighted changes in features and thus to allocate computing resources to more critical tasks. The attention mechanism has proven successful in recent years, particularly in the field of machine translation. The attention model consists of three important components^31^, namely Key, Value, and Query. When the attention mechanism selects the information in the input source, it first uses Query to question the pair of < Key, Value > . For a Query, the attention mechanism first calculates the correlation between the Query and each Key (i.e., the score), before using the softmax function to normalize the score to obtain the relative weight of each < Key, Value > . Finally, the value is weighted so as to obtain information related to the current Query, that is, the Attention Value. In this paper, Query, Key, and Value are abbreviated as *Q*, *K,* and *V*, respectively.

The Self-attention mechanism used in the paper is a special case of the attention mechanism in that the Query, Key, and Value come from the same set of inputs, $Q=K=V$, which ignores the distance between words and calculates the dependency relationship directly to learn the internal structure of a sentence. The self-attention layer receives the output $H=\left[{h}_{1},{h}_{2},\dots ,{h}_{t}\right]$ of the two-layer Bi-LSTM, computes the corresponding Query, Key, and Value, and then computes their attention scores. It then performs a weighted summation of the attention scores to derive the self-attention vector of the current input $O=\left[{o}_{1},{o}_{2},\dots ,{o}_{t}\right]$. The procedure for calculating the self-attention is as follows:

*Step 1* Randomly initialize the ${w}^{q}$, ${w}^{k}$, and ${w}^{v}$ weight matrices for each input *Q*, *K,* and *V* respectively. The three weight matrices are of equal size and are assumed to all be ${2d}_{h}\times m$ matrices, with *m* being the dimensions of *Q*, *K,* and *V*.

*Step 2* The ${w}^{q}$, ${w}^{k}$, and ${w}^{v}$ weight matrices are multiplied by each input vector ${h}_{i}$ to obtain $Q=\left[{q}_{1},{q}_{2},\dots ,{q}_{m}\right]$, $K=\left[{k}_{1},{k}_{2},..,{k}_{m}\right]$, and $V=\left[{v}_{1},{v}_{2},\dots ,{v}_{m}\right]$ corresponding to the vectors, respectively, where ${q}_{i}$, ${k}_{i}$, and ${v}_{i}$ are the Query, Key, and Value of the ${h}_{i}$-th vector in the input sequence.

10 $$Q=H{w}^{q},{w}^{q}\in {R}^{{2d}_{h}\times m}$$

11 $$K=H{w}^{k},{w}^{k}\in {R}^{{2d}_{h}\times m}$$

12 $$V=H{w}^{v},{w}^{v}\in {R}^{{2d}_{h}\times m}$$

where $H\in {R}^{t\times {2d}_{h}}$,${h}_{i}\in {R}^{{2d}_{h}}$, *t* denotes the length of the sentence, ${d}_{h}$ is the size of the LSTM hidden layer.

*Step 3* Calculate the self-attention vector:

13 $$O=attention\left(Q,K,V\right)=softmax\left(\frac{Q{K}^{T}}{\sqrt{{d}_{K}}}\right)\cdot V,O\in {R}^{t\times m}$$

where through dividing by $\sqrt{{d}_{K}}$ after the dot-product calculation, this method can obtain a more stable gradient.

### Word sense induction module

The WSI module utilizes contextual feature information to automatically perceive and classify the senses of polysemous words using a *K*-means algorithm, which was improved by optimizing the DPC algorithm based on cosine similarity, to construct a word sense vector for each sense. When constructing word sense embeddings of polysemous words through a WSI task, three significant factors are considered: (1) how to decide the number of senses that should be applied to each polysemous word; (2) how to group similar instances; and (3) how to construct an embedding representation for each sense.

For factor (1), the contextual feature extraction module extracts contextual information from each polysemous word instance to automatically perceive and divide the sense of polysemous words using the optimized DPC algorithm.

In factor (2), we used clustering algorithms that assumed that each cluster represents a sense of a word. Our study evaluated several clustering algorithms, including *K*-means^32^, mean shift^33^, DBSCAN^34^, spectral clustering^35^, and agglomerative clustering^36^. *K*-means, the distance-based clustering algorithm, performed slightly better and was therefore chosen for this study.

As for factor (3), the cluster center represents the word sense embeddings corresponding to each polysemy word sense.

Considering the three factors above, we propose the CDPC + *K*-means method in this module. First, the output of the contextual feature extraction module is used as the input of the optimized DPC algorithm based on cosine similarity to determine the number of senses of polysemous words, which determines the number of clusters for the subsequent *K*-means. Then, the *K*-means algorithm is applied to clusters of polysemy word instances. Finally, the cluster center represents the word sense embeddings corresponding to each polysemy word sense.

#### DPC

Rodriguez and Laio^37^ proposed a DPC algorithm in 2014. Since then, a variety of DPC variant algorithms have been developed^38^. The core idea of the DPC algorithm is founded on the assumption that, in a dataset with an arbitrary data distribution, clustering centers tend to be surrounded by objects with a lower local density and are relatively distant from objects with a higher local density in a dataset with an arbitrary data distribution. Based on this assumption, the DPC algorithm needs to calculate two indicators: one is the local density of data objects, and the other is the relative distance of data objects, both of which need to be calculated by the distance between data objects. Then, a cluster center decision graph is constructed by calculating the local density and relative distances, which are used to select the cluster centers. Finally, clustering is completed by assigning the remaining data objects to the nearest cluster centers.

In general, DPC is effective at detecting arbitrarily shaped clusters, is very simple to use, and has a high level of robustness. By using the decision graph without prior knowledge, the DPC is capable of locating the cluster center quickly and without requiring an iterative process and many parameters. The hypothetical dataset $\mathrm{X}=\left\{{x}_{1},\dots ,{x}_{i},\dots ,{x}_{n}\right\}, {x}_{i}=\left[{x}_{i1},...,{x}_{im}\right]$, in which *n* is the number of points and *m* is the dimension of the data. The local density ${\rho }_{i}$ of any point ${x}_{i}$ is **Definition 1:**

14 $${\rho }_{i}=\sum_{i\ne j}\tau \left({d}_{ij}-{d}_{c}\right)$$

where $$\tau \left(x\right)=\left\{\begin{array}{c}1,x<0\\ 0,x\ge 0\end{array}\right.$$, ${d}_{ij}={\Vert {x}_{i}-{x}_{j}\Vert }_{2}$ represents the Euclidean distance between ${x}_{i}$ and ${x}_{j}$, and ${d}_{c}$ is the truncation distance. If ${d}_{ij}-{d}_{c}<0$, then $x\left({d}_{ij}-{d}_{c}\right)=1$; else $x\left({d}_{ij}-{d}_{c}\right)=0$.

Equation (14), shows that ${\rho }_{i}$ represents all data objects where ${x}_{i}$ does not exceed ${d}_{c}$. Furthermore, relative distance ${\delta }_{i}$ refers to the distance at which the local density exceeds ${x}_{i}$ and the distance from its nearest point is as follows:

15 $${\delta }_{i}=\left\{\begin{array}{c}\mathrm{min}\left({d}_{ij}\right),{\rho }_{j}>{\rho }_{i}\\ max\left({d}_{ij}\right),i\ne j,point \;{x}_{i} \; with\, highest\, density\end{array}\right.$$

The main steps of DPC are to first calculate the local density and high-density nearest neighbor distance of all points. Then, a decision graph is constructed using local density and nearest neighbor distances, and the point with the greatest local density and the greatest nearest neighbor distance is selected as the cluster center. In the next step, the remaining points are allocated to clusters where their closest neighbors with a high density are located. Finally, if the distance between any two points in a cluster is less than ${d}_{c}$, then the point is a boundary point, and the point with the highest local density among the boundary points is defined as ${\rho }_{b}$. A cluster core is a point whose local density exceeds ${\rho }_{b}$, while an object with a local density equal to or less than ${\rho }_{b}$ is considered an outlier.

#### Optimized DPC

It has been demonstrated experimentally that cosine similarity is more effective as a measure of similarity for text clustering than Euclidean distance^39^. Therefore, we redefined Definition 1 in terms of cosine similarity. An optimized DPC algorithm based on cosine similarity is called CDPC.

##### Definition 2

local density ${\rho }_{i}$ based on cosine similarity:

When two vectors are in space $X=\left({x}_{1}, {x}_{2},\dots ,{x}_{k}\right)$ and $Y=\left({y}_{1}, {y}_{2},\dots ,{y}_{k}\right)$, their cosine similarity is defined as the cosine of the angle between them:

16 $$\mathrm{cos}(i,j)=\frac{\sum_{m=1}^{k}{x}_{m}{y}_{m}}{\sqrt{\sum_{m=1}^{k}{x}_{m}^{2}}\sqrt{\sum_{m=1}^{k}{y}_{m}^{2}}}=\frac{\sum_{m=1}^{k}{x}_{m}{y}_{m}}{\Vert x\Vert \cdot \Vert y\Vert }$$

17 $${\rho }_{i}={\sum }_{i\ne j}x\left(\mathrm{cos}\left(i,j\right)-\mathrm{cos}c\right)$$

In this equation, $x\cdot y$ is the dot product of the x and y vectors, $\mathrm{cos}\left(i,j\right)$ is the cosine similarity between *v*_*i*_ and *v*_*j*_, and $\mathrm{cos}c$ is the cut-off distance, which needs to be manually set to the nearest neighbor number of the sample at approximately 1–2% of the total size of the entire dataset. Based on Eq. (17), the more points *i* within $\mathrm{cos}c$, the greater the local density $\rho$.

##### Definition 3

relative distance $${\delta }_{i}$$:

18 $${\delta }_{i}=\left\{\begin{array}{c}\mathrm{min}\left(\mathrm{cos}\left(i,j\right)\right),{\rho }_{j}>{\rho }_{i}\\ max\left(\mathrm{cos}\left(i,j\right)\right),i\ne j,point {x}_{i} with\, highest\, density\end{array}\right.$$

**Figure 3:**
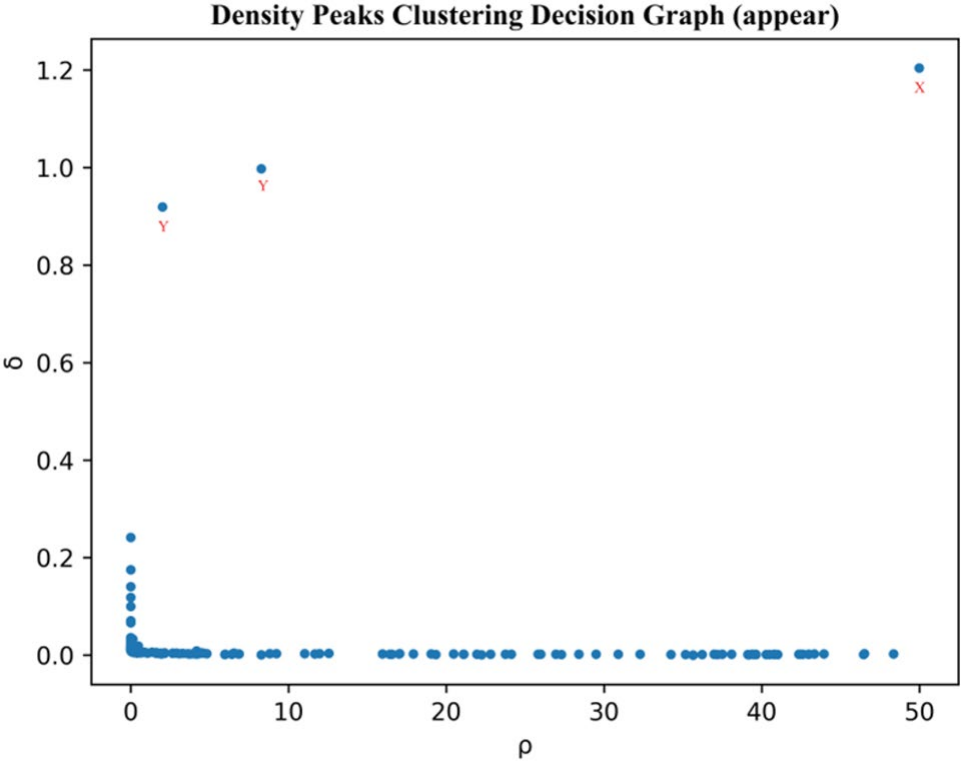
Decision graph for polysemous “appear” using optimized DPC algorithm based on cosine similarity.

**Figure 4:**
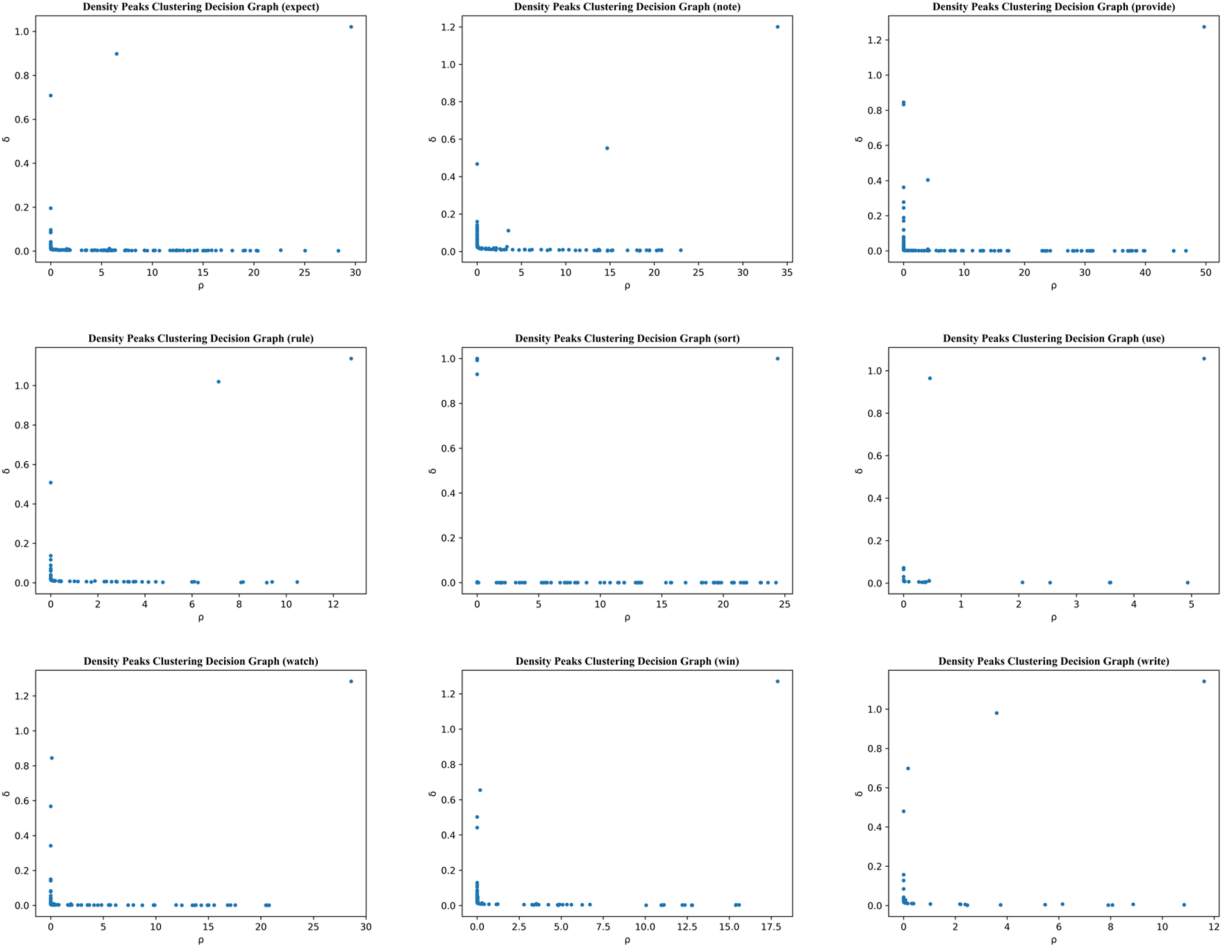
The decision graph for the polysemous word using the optimized DPC algorithm based on cosine similarity.

A decision graph (${\rho }_{i}$ is the horizontal coordinate and ${\delta }_{i}$ is the vertical coordinate) can now be constructed using the local density and nearest neighbor distance of the high density. The decision graph is a very effective tool for analyzing data structures. The DPC algorithm uses the decision graph to quickly identify cluster centers and then estimate the number of clusters. Figure 3 shows the decision graph for the polysemous word “appear” using the optimized DPC algorithm based on cosine similarity. As shown in Fig. 4, the point in the upper right corner of the graph has a higher ρ and δ, such as X, which is the most consistent with the DPC assumption. Not only does the point have a high local density, but it is also is far away from other high-density points. Hence, X can be chosen as the cluster center. The points in the upper left corner have a higher δ value and a smaller ρ value, such as Y, and their own density is higher than many points in the left lower corner, so they can be considered the cluster center. The points in the lower left and right corners are non-cluster center points. Based on Fig. 3, it can be estimated that the polysemous word “appear” has three sense clusters.

### Output

Following the determination of the number of clusters, *K*-means clustering was used to construct word sense clusters. Instances of the same sense are grouped into independent clusters. Clusters represents word senses, and cluster center vectors *C* represent word sense vectors for each polysemous word sense, as shown below:

19 $${c}_{j}=\frac{{X}_{1}+{X}_{2}+\cdots +{X}_{n}}{\left|{X}_{1}\right|+\left|{X}_{2}\right|+\cdots +\left|{X}_{n}\right|}=\left({w}_{1}{\prime},{w}_{2}{\prime},\cdots ,{w}_{k}{\prime}\right)$$

where *n* refers to the number of samples in word sense cluster *j*; ${X}_{i}$ represents each individual data object in cluster *j*; and $K$ represents the dimension of the output vector of the contextual feature extraction module.

## Experiment

### Dataset description

For the evaluation of WSI and the constructed word sense embeddings of polysemous words, we utilized the SemEval-2007 Task 17: English Lexical Sample, which provides instances of short texts that represent the context of polysemous words. The lexical sample consisted of 100 polysemous words with varying degrees of polysemy (ranging from 1 to 13) in 27,132 instances. We selected 10 polysemous words from the following list, whose polysemy ranged from 2 to 4. The dataset on polysemy is summarized in Table 1.

**Table 1:** The summary of the polysemy data set.

Polysemous words	Number of senses	Number of instances
Appear	3	233
Expect	3	134
Note	3	130
Provide	3	114
Rule	3	59
Sort	4	146
Use	2	23
Watch	4	98
Win	4	74
Write	4	44

### Comparison systems and evaluation metrics

We compared the impact of different similarity measures and different clustering algorithms in the cluster analysis on cluster quality. Similarity measures included Euclidean distance, cosine similarity, Pearson correlation, and KL-divergence. The clustering algorithms included *K*-means, mean shift, DBSCAN, spectral clustering, and agglomerative clustering.

The results of the clustering were evaluated using five metrics: clustering number, adjusted rand index (ARI), adjusted mutual information (AMI), V-measure, and silhouette coefficient. For ARI, AMI, and the silhouette coefficient, the value range was [− 1, 1], while for the V-measure, the value range was [0, 1]. For ARI, AMI, V-measures, and silhouette coefficients, each is evaluated such that the larger the value, the closer to one and the better the clustering effect. Clustering performance is better the closer the clustering number is to the number of real clusters.

### Experimental results and analysis

The method is comprised of a two-layer Bi-LSTM containing 50 nodes each in the forward and backward LSTM layers. The embedding dimension is 100, the learning rate is 0.0003, the batch size is 16, and the epoch is 50. This experiment specifies a maximum sentence length of 25 and trains the model using the Adam optimization method. In order to prevent overfitting of the model, a dropout method was applied to the input word vector as well as the attention network during the training process. The dropout was adjusted to 0.3. The model is programmed in Python, and is built using the TensorFlow framework. Table 2 shows the number of clusters created by the DPC algorithm for different similarity measures.

**Table 2:** Clusters created by the DPC algorithm are based on different similarity measures.

Polysemous words		Similarity measures	Clusters created by DPC	Number of real clusters
Appear	Euclidean distance		1	3
	Cosine similarity		**3**	3
	Pearson correlation		2	3
	KL-Divergence		1	3
Expect	Euclidean distance		2	3
	Cosine similarity		**3**	3
	Pearson correlation		2	3
	KL-Divergence		2	3
Note	Euclidean distance		4	3
	Cosine similarity		**3**	3
	Pearson correlation		2	3
	KL-Divergence		2	3
Provide	Euclidean distance		1	3
	Cosine similarity		**3**	3
	Pearson correlation		4	3
	KL-Divergence		1	3
Rule	Euclidean distance		2	3
	Cosine similarity		**3**	3
	Pearson correlation		2	3
	KL-Divergence		2	3
Sort	Euclidean distance		1	4
	Cosine similarity		**4**	4
	Pearson correlation		2	4
	KL-Divergence		1	4
Use	Euclidean distance		1	2
	Cosine similarity		**2**	2
	Pearson correlation		1	2
	KL-Divergence		2	2
Watch	Euclidean distance		1	4
	Cosine similarity		**3**	4
	Pearson correlation		3	4
	KL-Divergence		1	4
Win	Euclidean distance		2	4
	Cosine similarity		**3**	4
	Pearson correlation		3	4
	KL-Divergence		2	4
Write	Euclidean distance		2	4
	Cosine similarity		**3**	4
	Pearson correlation		3	4
	KL-Divergence		2	4

According to the literature^37^, the cut-off distance of the DPC parameter should be set at 1% to 2% of the total dataset size. Based on this valuable principle, we were able to determine the correct number of class clusters in our experiments. This parameter was not found to have a significant impact on the algorithm's results. We tested several values between 1 and 2% separately. The clustering results showed only a slight variation, indicating that the parameters in the DPC algorithm were robust. Table 2 shows that the optimized DPC algorithm based on cosine similarity (CDPC) can accurately determine the number of clusters, except for “watch”, “win”, and “write”. In comparison to other similarity measures, the CDPC clustering of “watch”, “win”, and “write” provided the closest estimates of the number of real clusters. Moreover, the experimental results indicate that clustering accuracy decreases as the number of clusters increases. Figure 4 shows the decision graph for the polysemous word using the optimized DPC algorithm based on cosine similarity. In conclusion, the CDPC algorithm can effectively identify polysemous word clusters. Furthermore, the clustering performance is superior to that of the traditional DPC algorithm, which is based on Euclidean distance.

For each clustering algorithm, the ARI results are presented in Table 3, the AMI results in Table 4, the V-measure results in Table 5, and the silhouette coefficient results in Table 6.

**Table 3:** The ARI of different clustering algorithms on polysemous words.

	CDPC + *K*-means (Ours)	Mean shift	DBSCAN	CDPC + spectral clustering	CDPC + agglomerative clustering
Appear	**0.87**	0.87	0.87	0.86	0.87
Expect	**0.96**	0.95	0.9	0.95	0.96
Note	0.84	**0.85**	0.84	0.84	0.81
Provide	**0.89**	0.89	0.89	0.85	0.89
Rule	0.94	**0.95**	0.94	0.94	0.94
Sort	0.85	**0.86**	0.85	0.79	0.86
Use	**0.84**	0.81	0.84	0.84	0.84
Watch	0.83	**0.85**	0.83	0.78	0.83
Win	**0.76**	0.70	0.72	0.63	0.70
Write	0.73	**0.74**	0.71	0.72	0.67

Significant values are in bold.

**Table 4:** The AMI of different clustering algorithms on polysemous words.

	CDPC + *K*-means (Ours)	Mean shift	DBSCAN	CDPC + spectral clustering	CDPC + agglomerative clustering
Appear	**0.76**	0.7	0.76	0.74	0.76
Expect	**0.91**	0.87	0.82	0.82	0.82
Note	0.75	0.74	0.75	**0.76**	0.71
Provide	**0.81**	0.79	0.81	0.73	0.81
Rule	0.92	**0.93**	0.92	0.92	0.93
Sort	0.72	**0.75**	0.70	0.70	0.73
Use	0.78	**0.80**	0.78	0.78	0.78
Watch	0.75	**0.76**	0.70	0.69	0.70
Win	0.65	**0.68**	0.65	0.56	0.61
Write	**0.74**	0.74	0.72	0.74	0.66

Significant values are in bold.

**Table 5:** The V-measure of different clustering algorithms on polysemous words.

	CDPC + K-means (Ours)	Mean shift	DBSCAN	CDPC + spectral clustering	CDPC + agglomerative clustering
Appear	**0.76**	0.76	0.76	0.74	0.76
Expect	0.83	**0.87**	0.83	0.83	0.83
Note	0.75	**0.77**	0.75	0.77	0.72
Provide	**0.81**	0.80	0.81	0.74	0.81
Rule	**0.93**	0.93	0.93	0.93	0.93
Sort	0.73	**0.76**	0.71	0.71	0.74
Use	0.79	**0.82**	0.79	0.79	0.79
Watch	0.72	**078**	0.72	0.71	0.72
Win	0.68	**0.71**	0.68	0.58	0.63
Write	0.75	**0.76**	0.75	0.76	0.68

Significant values are in bold.

**Table 6:** The silhouette coefficient of each clustering algorithm.

	CDPC + K-means (Ours)	Mean shift	DBSCAN	CDPC + spectral clustering	CDPC + agglomerative clustering
Appear	**0.82**	0.82	0.82	0.81	0.82
Expect	**0.95**	0.42	0.91	0.93	0.95
Note	**0.79**	− 0.1	0.79	0.65	0.60
Provide	**0.95**	0.88	0.95	0.93	0.95
Rule	**0.95**	0.60	0.95	0.95	0.95
Sort	**0.83**	0.52	0.81	0.81	0.83
Use	**0.88**	0.36	0.88	0.88	0.88
Watch	**0.91**	− 0.87	0.91	0.90	0.91
Win	0.61	0.62	**0.65**	0.60	0.61
Write	**0.61**	0.57	0.55	0.58	0.56

Significant values are in bold.

The experimental algorithms were run 10 times, and the results were averaged. The ARI, AMI, and silhouette coefficient values for the proposed CDPC + *K*-means algorithm were all significantly higher than those for the other four clustering algorithms. Compared with mean shift, DBSCAN, CDPC + spectral clustering and CDPC + agglomerative clustering, the ARI values in the 10 polysemous word datasets used increased by 0.5%, 1.4%, 3.6%, and 1.6%, respectively; AMI values increased by 0.4%, 2.3%, 4.5%, and 3.6%, respectively; and silhouette coefficient values increased by 54%, 0.1%, 3.1%, and 2.9%, respectively. In the V-measure metric, the mean shift performed well, but it was not much different from CDPC + *K*-means. Overall, the CDPC + *K*-means algorithm presented in this paper shows superior clustering performance compared to other clustering algorithms.

Based on the experiments in this paper, we conclude that the proposed two-layer bidirectional LSTM, combined with the self-attention mechanism, can extract more contextual information from polysemous word instances, providing high-quality text information for subsequent WSI. CDPC was more effective at identifying the number of sense clusters of polysemous words, and CDPC + *K*-Means exhibited superior clustering performance in WSI.

**Figure 5:**
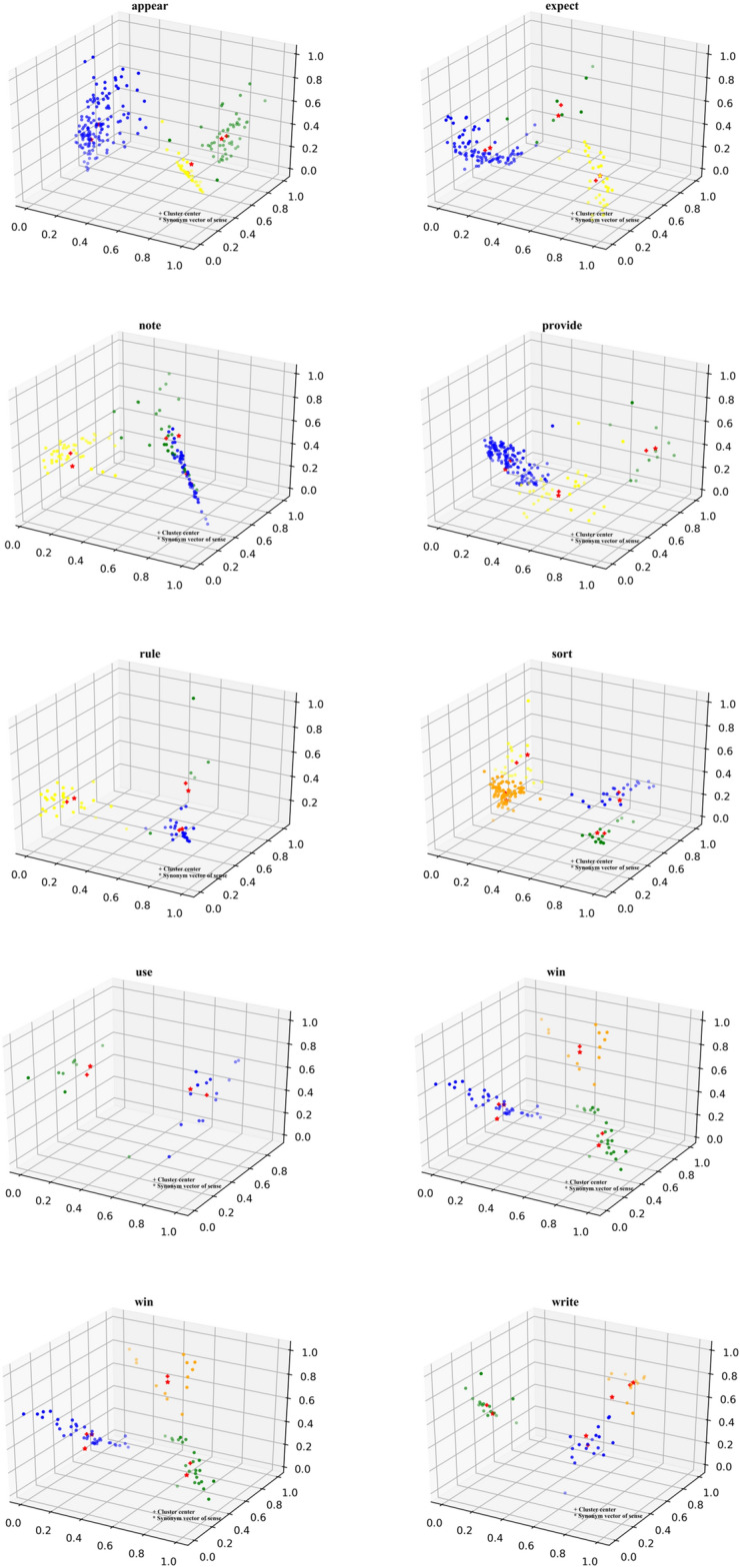
The results of CDPC + *K*-means on polysemous words clustering and synonym embeddings after dimensionality reduction.

As WordNet is an LKB containing synonym sets, where a word has multiple senses, there will also be multiple synsets. Our evaluation of the quality of the constructed word sense embeddings is based on the synsets of polysemous words. To evaluate the quality of the word sense embeddings of the constructed polysemous words, we used the synonym embeddings of each word sense of the polysemous words in WordNet. The results of CDPC + *K*-means on polysemous words clustering and synonym embeddings after dimensionality reduction were visualized in three-dimensional space, as shown in Fig. 5. We used the Isomap^40^ dimensionality reduction method to reduce the dimensionality of high-dimensional data^41^.

In the three-dimensional graph, different colors represent different clusters. Each red “ + ” represents the center of a class cluster, which is an embedding of each word sense of the polysemous word we constructed. The red “*” represents synonyms embeddings for each word sense of the polysemous word in WordNet. We can observe from the clustering results graph that the word sense embeddings are analogous to synonym embedding for word sense. This indicates that the word sense embeddings we constructed can accurately reflect each word sense. As a result, the sense of the polysemous word is more accurately expressed. In addition, the semantic information contained in the vector has greater clarity, and it is no longer a mixture of multiple senses.

As for “watch”, “win”, and “write”, the predicted number of clusters was smaller than the true number of clusters. The clustering results show that the word sense that is not predicted is missed because it is very similar to one of the predicted senses. This is because the synonym embedding these two senses is very close in the graph. For instance, “write” has a synonym of “author compose” and another synonym of “compose.” The example sentences of these two senses are “Gertrude Stein later wrote a book on Picasso” and “I think anything that is well written that's important.” The senses of “write” are reflected in the two synonyms, and the two example sentences are also very similar. As WordNet is fine-grained, so, too, is word sense tagging. Using CDPC + *K*-means clustering, these two senses are grouped together into one category, which results in an underestimation of the number of clusters.

## Conclusion

We proposed a word sense embeddings construction method based on WSI, which uses an unsupervised method to achieve dynamic word sense perception and improves the word sense embeddings construction process. As a result, word sense embeddings contain more accurate word sense information and achieve an improved quality of multi-sense word sense embeddings. The method mainly includes two modules: a polysemous word context information extraction module and an improved *K*-means algorithm. Contextual information extraction combines the two-layer Bi-LSTM with a self-attention mechanism to compensate for the limited ability of the Bi-LSTM to highlight significant features in long sequences; a *K*-means algorithm improved by optimizing the DPC algorithm based on cosine similarity is more appropriate for clustering polysemy texts. The experimental results indicate that the proposed method in this paper can effectively improve the quality of word sense embeddings.

## Data Availability

The datasets analyzed during the current study are available in the SemEval-2007 repository, https://semeval2.fbk.eu.
